# Cohesin mutations in myeloid malignancies: underlying mechanisms

**DOI:** 10.1186/2162-3619-3-13

**Published:** 2014-05-08

**Authors:** Bryony Leeke, Judith Marsman, Justin M O’Sullivan, Julia A Horsfield

**Affiliations:** 1 Department of Pathology, Dunedin School of Medicine, The University of Otago, P.O. Box 913, Dunedin, New Zealand; 2 Liggins Institute, The University of Auckland, Private Bag 92019, Auckland 1142, New Zealand

**Keywords:** Cohesin, Mutation, RUNX1, Myeloid, Leukemia, Transcription

## Abstract

Recently, whole genome sequencing approaches have pinpointed mutations in genes that were previously not associated with cancer. For Acute Myeloid Leukaemia (AML), and other myeloid disorders, these approaches revealed a high prevalence of mutations in genes encoding the chromosome cohesion complex, cohesin. Cohesin mutations represent a novel genetic pathway for AML, but how AML arises from these mutations is unknown. This review will explore the potential mechanisms by which cohesin mutations contribute to AML and other myeloid malignancies.

## Introduction

The development and pathology of acute myeloid leukemia (AML) can be caused by a number of genetic alterations, although the molecular basis of AML is not yet thoroughly understood. Chromosomal translocations and variations such as t(15;17), t(8:21), inv(16), t(9;21), t(9;11) are characteristic of AML, and suggest that genetic events play a key role in leukemogenesis [1]. However, nearly 50% of AML cases have a normal karyotype and lack major chromosome abnormalities. In an effort to elucidate the genetic basis of these cases, next-generation genome sequencing methods have been successfully used in recent years to identify many novel leukemogenic genes [2]. From these analyses, recurrent mutations in genes encoding subunits of the cohesin complex emerged in AML genomes. Several studies have now revealed that mutations in the cohesin complex are strongly associated with AML, and furthermore, that cohesin mutations are also found at high frequency in other related myeloid malignancies. Cohesin mutations could therefore represent a potential new molecular mechanism underpinning oncogenesis. Cohesin has multiple functions, including roles in cell division, nuclear architecture, DNA damage repair, development and transcription, and these functions have been the subject of several comprehensive recent reviews [3-9]. In this concise, focused review, we will discuss molecular functions of cohesin that have potential to influence the etiology and progression of AML and other myeloid malignancies.

## Cohesin biology and cancer development

**Figure 1:**
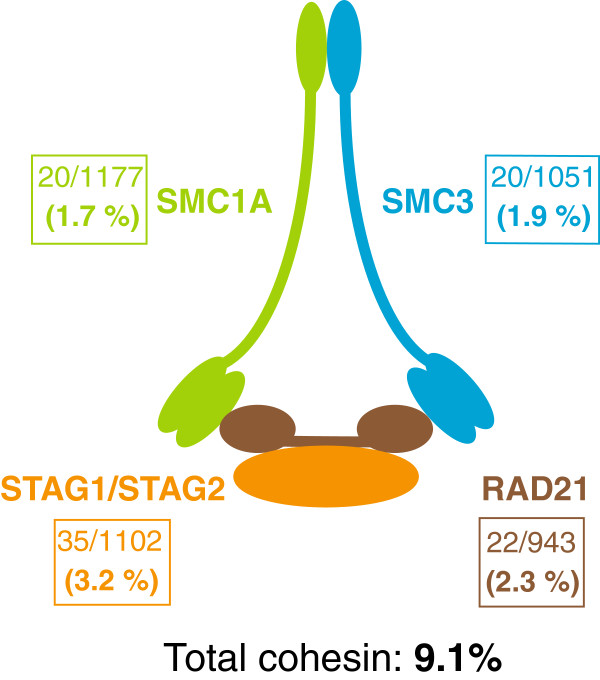
**Frequency of cohesin mutations in AML.** Cohesin is a multi-subunit protein complex that is involved in chromosome pairing, DNA repair and transcription regulation. Mutations within the individual protein components of cohesin occur at significant frequency in AML. Data from references 20–28 were combined to determine the mutation frequency (boxes) in each of the cohesin subunits (SMC1A, SMC3, STAG1/2, and RAD21). Details of mutations found in each study are presented in Table S1.

Cohesin is a large ring-shaped protein complex consisting of four major subunits: SMC1A, SMC3, RAD21 and STAG1/2 [7] (Figure 1). While best known for its role in mediating sister chromatid cohesion from S phase until M phase [7], cohesin also plays crucial roles in DNA damage repair and gene expression [10], human development [11] and cancer [12,13]. Cohesin mutations are found in several cancer types [14-16], however their contribution to oncogenesis is unclear with both overexpression and mutation of cohesin subunits being implicated in cancer. For example, overexpression of cohesin subunit RAD21 in breast cancer is associated with poor prognosis and resistance to chemotherapy [17]. Cohesin mutations must necessarily lead to reduced, but not absent function, since complete loss of cohesin function blocks mitosis and results in cell death [18,19]. Therefore, the cohesin mutations found in cancer are usually heterozygous or hypomorphic. The mechanisms by which cohesin mutations contribute to cancer probably involve multiple molecular pathways reflecting its non-mitotic molecular roles [12,13].

## Cohesin mutations in myeloid malignancies

The association of cohesin mutations with myeloid malignancy is particularly striking. Data from the Cancer Genome Atlas Research Network (TCGA) revealed that a significant proportion of AMLs had mutations in subunits of cohesin [20]. Somatic variants in cohesin genes were identified in 26/200 cases of AML subjected to exome or whole genome sequencing [20,21]. Sequencing of a separate set of AML samples by Welch *et al.* identified cohesin mutations in 7/108 cases [21]. Cohesin mutations occurred primarily in French-American-British (FAB) M1 and M2 cases in the TCGA cohort, and Welch *et al.* found cohesin mutations exclusively in M1 cases [21]. The predominance of cohesin lesions in the most immature forms of AML suggests they were initiating events rather than passenger mutations [21]. Cohesin mutations co-occurred with NPM1, DNMT3A, TET2, or RUNX1 mutations in 17/19 cases [21], implying cooperation with other leukemogenic pathways. Mutations in cohesin genes represented one of just nine categories of mutations thought to actively contribute to leukemogenesis [20]. Our calculation of the rate of cohesin mutations in AML using the TCGA data [20,21] and other published studies to date [22-28] indicates that the total rate of cohesin mutation in AML is around 9% (Figure 1, Table 1). Further details of the contributing studies can be found in the accompanying supplementary table (see Additional file 1: Table S1).

**Table 1:** Key findings from selected studies identifying cohesin mutations in myeloid malignancies

**Myeloid disorder(s)**	**Cohesin mutation rate**	**Rationale and key findings**	**Ref #**
AML	26/200	200 *de novo* AML samples were submitted to whole-genome/exome sequencing. The genes identified as recurrently mutated were grouped into nine functional categories important for AML: the cohesin complex was one such category. Cohesin complex mutations were mutually exclusive.	[20]
	(13%)		
AML	7/108	Whole-genome sequencing (WGS) of 24 normal-karyotype M1 and M3 AML samples. Cohesin genes were only mutated in M1 samples. Cohesin mutations were mutually exclusive and were not associated with chromosomal instability.	[21]
	(6.5%)		
AML	23/389	Targeted sequencing of cohesin genes in 389 AML samples. Cohesin mutations significantly co-occurred with *NPM1* mutations. Allelic burden analysis suggested cohesin mutations occurred early in AML.	[24]
	(5.9%)		
AML	23/197	Targeted sequencing of 51 myeloid neoplasm candidate genes in 197 AML samples. Cohesin mutations were not associated with overall survival.	[26]
	(11.7%)		
AML	7/170	Targeted sequencing of AML candidate loci in 50 AML samples. *RAD21* mutations were present in all AML subtypes and were significantly associated with *RAS* mutations.	[27]
	(4.1%)		
AML	12/158	WGS of eight MDS and subsequent secondary-AML patient genomes. Targeted sequencing of 94 MDS/AML candidate loci. Each clone contained at least one mutation that recurs in MDS/AML. *STAG2* mutations significantly co-occurred with *RUNX1* mutations.	[28]
MDS	(7.6%)		
MDS	Approximately 15%	Targeted sequencing of 104 MDS/AML candidate genes in 944 MDS samples. 47 genes were recurrently mutated in MDS. 14 of these genes (including *STAG2)* could successfully predict survival-outcome risk groups. *STAG2* and *SMC1A* mutations were significantly associated with adverse patient outcome.	[29]
AML	65/610	Targeted sequencing of cohesin complex genes was undertaken in a cohort of 610 samples from various myeloid neoplasms. The core components of cohesin were significantly mutated. Cohesin mutations were present in the major tumor population in 15/20 available samples, indicating that cohesin mutations often occur as early events in oncogenesis.	[25]
MDS	(10.7%)		
CMML			
CML			
MPN			
TAM	39/86	WGS of the genomes of TAM, AMKL, and DS-AMKL patients. Progression to DS-AMKL required acquisition of further mutations, including *RAD21, STAG2, NRAS, CTCF, EZH2,* and *TP53*.	[30]
AMKL	(45.3%)		
DS-AMKL		Cohesin mutations were present at a much higher rate in DS-AMKL than AMKL. Allelic burden analysis suggested that cohesin mutations occurred early in DS-AMKL.	

The emergence of cohesin mutations in AML prompted Thol *et al.*[24] to sequence cohesin complex genes in 389 AML samples, yielding a total of 23 mutations (5.9%). Mutations in cohesin subunits were mutually exclusive, and most mutations were found in karyotypically normal samples. A strong correlation was observed between mutations in cohesin and the known AML-associated gene nucleophosmin (*NPM1)*, with *NPM1*-mutated patients twice as likely to also harbor a cohesin mutation compared with *NPM1*-normal. Cohesin mutation status was not prognostically informative, nor did it correlate with any differences in clinical features. Allelic burden analysis suggested that cohesin mutations occurred as an early event during leukemogenesis [24].

While most evidence for cohesin mutations in myeloid leukemia currently comes from AML, cohesin mutation is also implicated in related myeloid disorders. For example, Kon *et al.*[25] reported frequent mutations in cohesin components in a variety of myeloid neoplasms, including AML, myelodysplastic syndromes (MDS), chronic myelomonocytic leukemia (CMML), chronic myelogenous leukemia (CML) and classical myeloproliferative neoplasms (MPN). Deep sequencing revealed that the majority of cohesin mutations existed in the major tumor populations, indicating they arose early in neoplasia. Strikingly, despite cohesin’s known role in sister chromatid cohesion, myeloid malignancies with cohesin mutations were no more likely to be aneuploid than leukemias harboring other mutations [25]. Kon *et al.* conclude that, owing to their early origin and frequency in myeloid neoplasms, cohesin mutations actively contribute to leukemogenesis [25].

Further evidence of cohesin’s involvement in myeloid malignancies emerged from a recent study by Haferlach *et al.* showing that approximately 15% of patients with MDS harbor cohesin mutations [29]. The high proportion of cohesin mutations in MDS, combined with the fact that STAG2 and SMC1A mutations were significantly associated with poor survival outcome, strongly suggests that cohesin mutation is central to the development and prognosis of MDS [29].

Yoshida *et al.* identified a striking association of cohesin mutation with another myeloid dysplasia, DS-AMKL [30]. Down’s Syndrome (DS) patients can present with transient abnormal myelopoiesis (TAM) that is self-limiting in most cases. TAM is a myeloid proliferation resembling AML, and 10% of TAM progresses to non self-limiting acute megakaryoblastic leukemia (AMKL) in DS patients (DS-AMKL). Deep sequencing revealed that 53% of DS-AMKL samples had acquired cohesin mutations that were not found in somatic cells or the original TAM [30]. The high frequency of lesions in cohesin raises the strong possibility that cohesin mutation is instrumental to progression to DS-AMKL.

Despite the prevalence of cohesin mutations in myeloid dysplasia, the exact mechanism by which cohesin lesions contribute to cancer development is unclear. Accumulating evidence argues that cohesin mutation is an early event in myeloid oncogenesis. Welch *et al.* and TCGA showed that cohesin mutations mainly occur in the most immature AML subtypes [20,21]; clonal analysis by Kon *et al.*[25] and allelic burden analysis by Thol *et al.*[24] suggest that cohesin mutations occur as early events in leukemogenesis. What is the mechanism by which these mutations lead to cancer? In solid tumors with cohesin mutations, chromosome instability and aneuploidy have been suggested as the mechanisms by which cohesin mutation facilitates neoplasia [16,31,32], although other evidence argues against this idea [33]. For myeloid malignancies, a clear theme is emerging: heterozygous cohesin mutations do not cause chromosome instability [21,22,24,25,30] (for details, see Additional file 1: Table S1). This suggests that, at least in myeloid cancers, it is cohesin’s non-mitotic roles that contribute to oncogenesis.

## Cohesin regulates gene transcription

**Figure 2:**
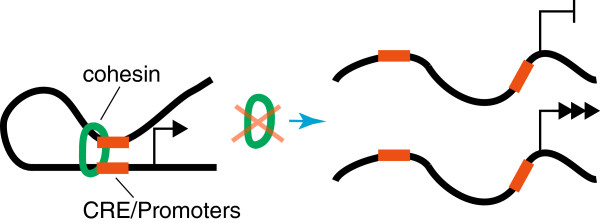
**Cohesin regulates gene expression by controlling CRE-promoter interactions.** CREs can regulate gene expression by physically contacting a promoter, but are often located at a distance (tens of kilobases and sometimes megabases) from the promoter. Cohesin is involved in the establishment and maintenance of CRE-promoter interactions and can thereby control gene expression. Loss of cohesin can lead to loss of CRE-promoter interactions, resulting in inappropriate gene repression, or gene activation.

Cohesin’s role in gene expression has been intensively investigated over the last 15 years [10]. Several examples of cohesin-dependent gene regulation have been found, including regulatory roles at developmental genes [5] and in stem cells [34]. One of the potential mechanisms by which cohesin regulates gene transcription is through mediating long-range communication events that form DNA loops, which regulate transcription [6]. Enhancers (which promote transcription) and insulators (which usually block transcription) are located in *c*onserved *r*egulatory *e*lements (CREs) on chromosomes, and need not be close to the gene(s) they regulate. Cohesin is thought to physically connect distant CREs with gene promoters, in a cell type-specific manner, to modulate transcriptional outcomes [6] (Figure 2). Therefore, mutations in cohesin could impede cohesin binding to CREs, thereby altering their interaction with promoters, and subsequently gene activity. Similarly, mutations in the CREs that affect cohesin binding could alter transcription of the gene target(s) of that CRE.

In addition to connecting CREs with promoters, cohesin has an important role in organizing global genomic architecture. Cohesin binding of DNA together with CCCTC-binding factor (CTCF) helps to partition the genome into megabase-sized regions known as *t*opologically *a*ssociated *d*omains (TADs) [35-37]. TADs are demarcated by boundaries that are characterized by the presence of cohesin and CTCF, housekeeping genes, tRNAs and short interspersed element (SINE) retrotransposons [35]. Within TADs are regions of local chromosome interactions, which allow CREs to come into physical proximity with gene promoters to modulate gene expression [38-40]. While TAD boundaries are conserved between cell types, the chromosome interactions within TADs vary, and provide a means for enabling cell type-specific transcription [35,38,40].

**Figure 3:**
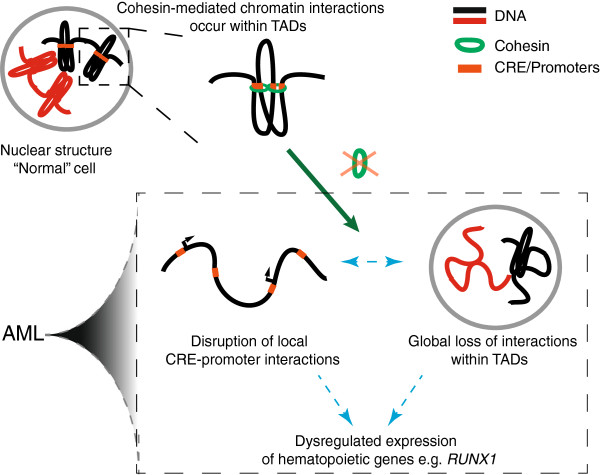
**Model for cohesin’s role in AML and other myeloid malignancies.** Cohesin has an important function in the nucleus: it mediates chromosome interactions within topologically associated domains (TADs). Within TADs, cohesin connects conserved regulatory elements (CREs) with promoters, thereby regulating gene transcription. When cohesin function is compromised by a heterozygous mutation, as in AML, this leads to loss of CRE-promoter communication at specific hematopoietic genes, such as *RUNX1*. The result is dysregulation of hematopoietic transcription programs, which could facilitate the development of AML. In addition, loss of tissue-specific sub-domain structures affects the global hematopoietic transcription program.

Although cohesin and CTCF frequently colocate on chromosomes [41-43], they appear to have distinct roles in genome architecture [40,44,45]. Cohesin influences gene expression by coordinating interactions between CREs and promoters within TADs [38,40], while CTCF is important for preventing interactions between TADs [40]. Cohesin deficiency reduces the number of chromosome interactions within TADs and leads to altered expression of many genes [38-40]: different genes to those dysregulated upon CTCF depletion [40]. Because of the cell type specificity of CRE-promoter interactions within TADs, cohesin deficiency could result in an abnormal transcriptional profile for a particular tissue type (Figure 3). Moreover, there are several genomic sites where cohesin binds exclusively of CTCF, in combination with tissue-specific transcription factors [44,45]. For example, in mouse primary liver cells and hepatocellular carcinoma cells (HepG2), CTCF-independent cohesin binding sites are associated with expression of liver-specific genes [44].

Strikingly, only a modest reduction in chromatin-bound cohesin is sufficient to cause changes in gene expression [46]. In human cells and mice, heterozygous mutations in the cohesin-loading factor NIPBL or in cohesin subunit SMC1A affect the expression of numerous genes [47,48]. In Drosophila, halving the gene dose of cohesin components robustly affects gene expression [49,50]. Therefore, leukemias with heterozygous cohesin mutations are also likely to be affected by the dysregulation of many genes.

## Altered cohesin function has potential to perturb hematopoietic gene expression

A number of hematopoietic transcription factors are regulated by cohesin binding to CREs or promoters. For example, the hematopoietic transcription factor TAL1 is regulated at the transcriptional level by a chromatin hub containing cohesin and CTCF [51]. In further examples, the *GATA2* gene contains an intronic +9.5 kb enhancer that is important for its expression [52], while the +85 *ERG* stem cell enhancer contains binding sites for a heptad of hematopoietic transcription factors and is thought to propagate a hematopoietic stem cell-like transcription profile [53]. Our survey of publically available ENCODE data revealed that cohesin binds both the *GATA2* and *ERG* enhancers in hematopoietic cells (K562). Therefore, it is possible that cohesin mutation could alter the activity of these enhancers and their target genes in a leukemogenic setting.

## *RUNX1* transcription is altered by cohesin deficiency

The developmental transcription factor RUNX1 plays a particularly important role in myeloid malignancies. RUNX1 function is central to early myeloid differentiation and is absolutely required for definitive hematopoiesis [54,55]. RUNX1 is involved in chromosomal translocations, such as t(12;21) in acute lymphoblastic leukemia in childhood and t(8;21) in acute myeloid leukemia, and is also targeted by point mutations and deletions [56]. Leukemic alterations of RUNX1 lead to abnormal protein function and thus dysregulation of RUNX1 target genes. The importance of RUNX1 function in hematopoiesis and leukemia has generated great interest in determining the factors that regulate its expression.

It is interesting that DS-AMKL leukemias contain three copies of the *RUNX1* gene (owing to trisomy 21), as well as having a remarkably high frequency of cohesin mutation (53%) [30]. Evocatively, data from zebrafish provided the first evidence that cohesin regulates tissue-specific *Runx1* transcription. In developing zebrafish embryos, a null mutation in the *rad21* subunit of cohesin blocked *runx1* expression in hematopoietic mesoderm, but not in Rohon-Beard neurons [57]. That cohesin ablation affected hematopoietic progenitors, but not neurons, indicates that the transcriptional role of cohesin is tissue-specific in hematopoietic precursors.

In mouse, a CRE enhancer resides in an intron between the P1 (distal) and P2 (proximal) promoters of *Runx1*. This enhancer*,* termed +23 [58] or alternatively, +24 [59], is active only in precursors of hematopoietic stem cells where *Runx1* is endogenously expressed [58,59]. Cohesin subunit Rad21 binds the *Runx1* + 23/24 mouse enhancer region, which is also conserved in human [60]. ENCODE data from the leukemia K562 cell line indicates that the equivalent human CRE/enhancer also recruits cohesin subunits, together with CTCF [60].

In zebrafish, Marsman *et al.* showed that cohesin depletion altered the activity of intronic *runx1* CREs [60]. Multiple binding sites were identified for cohesin and CTCF in the zebrafish *runx1* gene, coinciding with active CREs in the intron between P1 and P2. Cohesin and CTCF determine the spatial distribution of *runx1* transcripts in the zebrafish embryo at the onset of *runx1* expression, likely by controlling intronic CRE activity and CRE-promoter interactions. CTCF appears to restrict the expression pattern of *runx1*, consistent with insulator activity, while cohesin is necessary for its expression in a specific subpopulation of hematopoietic progenitors [57,60].

Interestingly, Marsman *et al.* also showed that siRNA knock down of cohesin (but not CTCF) in HL-60 myelocytic leukemia cells enhanced *RUNX1* transcription [60], indicating that cohesin’s transcriptional role is conserved in human cells. It is tempting to speculate that cohesin mutation leading to an increase in *RUNX1* transcription might exacerbate myeloid malignancies that already have excess *RUNX1*; for example, DS-AMKL [30].

In summary, it appears that cohesin has a crucial role in cell type-specific regulation of *Runx1*, likely by mediating interactions between CREs and the promoters of *Runx1*. In support of this idea, ChIA-PET data generated in K562 cells using RNA polymerase II demonstrated that the two promoters of human *RUNX1* are in physical proximity with each other, and with CREs in the intron between the two promoters [61]. It is not yet known whether these interactions regulate *RUNX1*, or whether they are cohesin-dependent. While formal proof of this kind of mechanism for cohesin regulation of *Runx1* is still to come, the link between cohesin mutation and spatiotemporal *Runx1* transcription may explain cohesin’s contribution to AML pathogenesis and other myeloid malignancies.

## Conclusions

Mutations in cohesin comprise a novel genetic pathway significantly associated with the development of AML and related leukemias. While several types of cancer do have cohesin mutations, most cancers also harbor many additional mutations in multiple gene categories [15]. By contrast, AML genomes contain relatively fewer mutations than other cancer types, with only 23 genes significantly mutated [20]. Four of these genes correspond to cohesin subunits [15,20], indicating that cohesin mutations are particularly important to the progression of AML.

Why do myeloid disorders have a high prevalence of cohesin mutations in particular? The answer could reside in cohesin’s potential to mediate global transcriptional activity in a way that is also exquisitely cell type-specific.

Evidence that cohesin regulates cell type-specific global gene transcriptional programs, and in particular, expression of the AML-associated transcription factor, *RUNX1*, could explain why cohesin mutations are so prevalent in myeloproliferative disorders. Perhaps correct differentiation along the myeloid pathway relies on accurate expression of key genes (such as *RUNX1*) that can only respond to a full complement of cohesin. When cohesin function is impaired, differentiation of myeloid precursors might be prevented, facilitating dysplasia. These notions support previous hypotheses that cohesin is likely to play an important role in hematopoiesis [57,62].

Remarkably, cohesin binds to a majority of cell type-specific transcription factor binding sites, even when transcription factors themselves are evicted during mitosis [63]. In this manner, cohesin binding may ‘bookmark’ transcription factor binding sites to re-establish transcriptional programs after cell division [63], including sites for hematopoietic transcription factors.

Further research will be necessary to understand exactly how cohesin functions in normal and abnormal hematopoiesis, and how cohesin mutations cooperate with other genetic events to progress leukemia.

## Abbreviations

AML: Acute myeloid leukemia; MDS: Myelodysplastic syndrome; CMML: Chronic myelomonocytic leukemia; CML: Chronic myelogenous leukemia; MPN: Myeloproliferative neoplasm; DS: Down’s Syndrome; TAM: Transient abnormal myelopoiesis; AMKL: Acute megakaryoblastic leukemia; CRE: Conserved regulatory element; CTCF: CCCTC-binding factor; TAD: Topologically associated domain; SINE: Short interspersed element.

## Competing interests

The authors declare they have no competing interests.

## Authors’ contributions

BL, JM, JO and JH wrote the article. BL researched the literature and prepared the tables. JO, JM, BL and JH prepared the figures. All authors read and approved the final manuscript.

## Supplementary Material

Additional file 1: Table S1 Comprehensive summary of cohesin mutations in myeloid malignancies. Click here for file

## References

[B1] Betz BL Hess JL Acute myeloid leukemia diagnosis in the 21st century Arch Pathol Lab Med 2010 134 1427 1433 20923295 10.5858/2010-0245-RA.1

[B2] Sanders MA Valk PJ The evolving molecular genetic landscape in acute myeloid leukaemia Curr Opin Hematol 2013 20 79 85 10.1097/MOH.0b013e32835d821c 23380602

[B3] Ball AR Jr Chen YY Yokomori K Mechanisms of cohesin-mediated gene regulation and lessons learned from cohesinopathies Biochim Biophys Acta 2014 1839 191 202 10.1016/j.bbagrm.2013.11.002 24269489 PMC3951616

[B4] Bose T Gerton JL Cohesinopathies, gene expression, and chromatin organization J Cell Biol 2010 189 201 210 10.1083/jcb.200912129 20404106 PMC2856913

[B5] Horsfield JA Print CG Monnich M Diverse developmental disorders from the one ring: distinct molecular pathways underlie the cohesinopathies Front Genet 2012 3 171 22988450 10.3389/fgene.2012.00171 PMC3439829

[B6] Merkenschlager M Odom DT CTCF and cohesin: linking gene regulatory elements with their targets Cell 2013 152 1285 1297 10.1016/j.cell.2013.02.029 23498937

[B7] Nasmyth K Haering CH Cohesin: its roles and mechanisms Annu Rev Genet 2009 43 525 558 10.1146/annurev-genet-102108-134233 19886810

[B8] Skibbens RV Colquhoun JM Green MJ Molnar CA Sin DN Sullivan BJ Tanzosh EE Cohesinopathies of a feather flock together PLoS Genet 2013 9 e1004036 10.1371/journal.pgen.1004036 24367282 PMC3868590

[B9] Wu N Yu H The Smc complexes in DNA damage response Cell Biosci 2012 2 5 10.1186/2045-3701-2-5 22369641 PMC3329402

[B10] Dorsett D Strom L The ancient and evolving roles of cohesin in gene expression and DNA repair Curr Biol 2012 22 R240 R250 10.1016/j.cub.2012.02.046 22497943 PMC3327610

[B11] Dorsett D Krantz ID On the molecular etiology of Cornelia de Lange syndrome Ann N Y Acad Sci 2009 1151 22 37 10.1111/j.1749-6632.2008.03450.x 19154515 PMC2733214

[B12] Rhodes JM McEwan M Horsfield JA Gene regulation by cohesin in cancer: is the ring an unexpected party to proliferation? Mol Cancer Res 2011 9 1587 1607 10.1158/1541-7786.MCR-11-0382 21940756

[B13] Xu H Tomaszewski JM McKay MJ Can corruption of chromosome cohesion create a conduit to cancer? Nat Rev Cancer 2011 11 199 210 10.1038/nrc3018 21326324

[B14] Taylor CF Platt FM Hurst CD Thygesen HH Knowles MA Frequent inactivating mutations of STAG2 in bladder cancer are associated with low tumour grade and stage and inversely related to chromosomal copy number changes Hum Mol Genet 2014 23 1964 1974 10.1093/hmg/ddt589 24270882 PMC3959811

[B15] Kandoth C McLellan MD Vandin F Ye K Niu B Lu C Xie M Zhang Q McMichael JF Wyczalkowski MA Leiserson MD Miller CA Welch JS Walter MJ Wendl MC Ley TJ Wilson RK Raphael BJ Ding L Mutational landscape and significance across 12 major cancer types Nature 2013 502 333 339 10.1038/nature12634 24132290 PMC3927368

[B16] Barber TD McManus K Yuen KW Reis M Parmigiani G Shen D Barrett I Nouhi Y Spencer F Markowitz S Velculescu VE Kinzler KW Vogelstein B Lengauer C Hieter P Chromatid cohesion defects may underlie chromosome instability in human colorectal cancers Proc Natl Acad Sci U S A 2008 105 3443 3448 10.1073/pnas.0712384105 18299561 PMC2265152

[B17] Xu H Yan M Patra J Natrajan R Yan Y Swagemakers S Tomaszewski JM Verschoor S Millar EK van der Spek P Reis-Filho JS Ramsay RG O'Toole SA McNeil CM Sutherland RL McKay MJ Fox SB Enhanced RAD21 cohesin expression confers poor prognosis and resistance to chemotherapy in high grade luminal, basal and HER2 breast cancers Breast Cancer Res 2011 13 R9 10.1186/bcr2814 21255398 PMC3109576

[B18] Vass S Cotterill S Valdeolmillos AM Barbero JL Lin E Warren WD Heck MM Depletion of Drad21/Scc1 in Drosophila cells leads to instability of the cohesin complex and disruption of mitotic progression Curr Biol 2003 13 208 218 10.1016/S0960-9822(03)00047-2 12573216

[B19] Rollins RA Korom M Aulner N Martens A Dorsett D Drosophila nipped-B protein supports sister chromatid cohesion and opposes the stromalin/Scc3 cohesion factor to facilitate long-range activation of the cut gene Mol Cell Biol 2004 24 3100 3111 10.1128/MCB.24.8.3100-3111.2004 15060134 PMC381657

[B20] Cancer Genome Atlas Research N Genomic and epigenomic landscapes of adult de novo acute myeloid leukemia N Engl J Med 2013 368 2059 2074 23634996 10.1056/NEJMoa1301689 PMC3767041

[B21] Welch JS Ley TJ Link DC Miller CA Larson DE Koboldt DC Wartman LD Lamprecht TL Liu F Xia J Kandoth C Fulton RS McLellan MD Dooling DJ Wallis JW Chen K Harris CC Schmidt HK Kalicki-Veizer JM Lu C Zhang Q Lin L O'Laughlin MD McMichael JF Delehaunty KD Fulton LA Magrini VJ McGrath SD Demeter RT Vickery TL The origin and evolution of mutations in acute myeloid leukemia Cell 2012 150 264 278 10.1016/j.cell.2012.06.023 22817890 PMC3407563

[B22] Rocquain J Gelsi-Boyer V Adelaide J Murati A Carbuccia N Vey N Birnbaum D Mozziconacci MJ Chaffanet M Alteration of cohesin genes in myeloid diseases Am J Hematol 2010 85 717 719 10.1002/ajh.21798 20687102

[B23] Jan M Snyder TM Corces-Zimmerman MR Vyas P Weissman IL Quake SR Majeti R Clonal evolution of preleukemic hematopoietic stem cells precedes human acute myeloid leukemia Sci Transl Med 2012 4 149 ra118 10.1126/scitranslmed.3004315 PMC4045621 22932223

[B24] Thol F Bollin R Gehlhaar M Walter C Dugas M Suchanek KJ Kirchner A Huang L Chaturvedi A Wichmann M Wiehlmann L Shahswar R Damm F Gohring G Schlegelberger B Schlenk R Dohner K Dohner H Krauter J Ganser A Heuser M Mutations in the cohesin complex in acute myeloid leukemia: clinical and prognostic implications Blood 2014 123 914 920 10.1182/blood-2013-07-518746 24335498

[B25] Kon A Shih LY Minamino M Sanada M Shiraishi Y Nagata Y Yoshida K Okuno Y Bando M Nakato R Ishikawa S Sato-Otsubo A Nagae G Nishimoto A Haferlach C Nowak D Sato Y Alpermann T Nagasaki M Shimamura T Tanaka H Chiba K Yamamoto R Yamaguchi T Otsu M Obara N Sakata-Yanagimoto M Nakamaki T Ishiyama K Nolte F Recurrent mutations in multiple components of the cohesin complex in myeloid neoplasms Nat Genet 2013 45 1232 1237 10.1038/ng.2731 23955599

[B26] Kihara R Nagata Y Kiyoi H Kato T Yamamoto E Suzuki K Chen F Asou N Ohtake S Miyawaki S Miyazaki Y Sakura T Ozawa Y Usui N Kanamori H Kiguchi T Imai K Uike N Kimura F Kitamura K Nakaseko C Onizuka M Takeshita A Ishida F Suzushima H Kato Y Miwa H Shiraishi Y Chiba K Tanaka H Comprehensive analysis of genetic alterations and their prognostic impacts in adult acute myeloid leukemia patients Leukemia 2014 doi:10.1038/leu.2014.55. [Epub ahead of print] 10.1038/leu.2014.55 24487413

[B27] Dolnik A Engelmann JC Scharfenberger-Schmeer M Mauch J Kelkenberg-Schade S Haldemann B Fries T Kronke J Kuhn MW Paschka P Kayser S Wolf S Gaidzik VI Schlenk RF Rucker FG Dohner H Lottaz C Dohner K Bullinger L Commonly altered genomic regions in acute myeloid leukemia are enriched for somatic mutations involved in chromatin remodeling and splicing Blood 2012 120 e83 e92 10.1182/blood-2011-12-401471 22976956

[B28] Walter MJ Shen D Shao J Ding L White BS Kandoth C Miller CA Niu B McLellan MD Dees ND Fulton R Elliot K Heath S Grillot M Westervelt P Link DC DiPersio JF Mardis E Ley TJ Wilson RK Graubert TA Clonal diversity of recurrently mutated genes in myelodysplastic syndromes Leukemia 2013 27 1275 1282 10.1038/leu.2013.58 23443460 PMC3736571

[B29] Haferlach T Nagata Y Grossmann V Okuno Y Bacher U Nagae G Schnittger S Sanada M Kon A Alpermann T Yoshida K Roller A Nadarajah N Shiraishi Y Shiozawa Y Chiba K Tanaka H Koeffler HP Klein HU Dugas M Aburatani H Kohlmann A Miyano S Haferlach C Kern W Ogawa S Landscape of genetic lesions in 944 patients with myelodysplastic syndromes Leukemia 2014 28 241 247 10.1038/leu.2013.336 24220272 PMC3918868

[B30] Yoshida K Toki T Okuno Y Kanezaki R Shiraishi Y Sato-Otsubo A Sanada M Park MJ Terui K Suzuki H Kon A Nagata Y Sato Y Wang R Shiba N Chiba K Tanaka H Hama A Muramatsu H Hasegawa D Nakamura K Kanegane H Tsukamoto K Adachi S Kawakami K Kato K Nishimura R Izraeli S Hayashi Y Miyano S The landscape of somatic mutations in Down syndrome-related myeloid disorders Nat Genet 2013 45 1293 1299 10.1038/ng.2759 24056718

[B31] Solomon DA Kim JS Bondaruk J Shariat SF Wang ZF Elkahloun AG Ozawa T Gerard J Zhuang D Zhang S Navai N Siefker-Radtke A Phillips JJ Robinson BD Rubin MA Volkmer B Hautmann R Kufer R Hogendoorn PC Netto G Theodorescu D James CD Czerniak B Miettinen M Waldman T Frequent truncating mutations of STAG2 in bladder cancer Nat Genet 2013 45 1428 1430 10.1038/ng.2800 24121789 PMC3875130

[B32] Guo G Sun X Chen C Wu S Huang P Li Z Dean M Huang Y Jia W Zhou Q Tang A Yang Z Li X Song P Zhao X Ye R Zhang S Lin Z Qi M Wan S Xie L Fan F Nickerson ML Zou X Hu X Xing L Lv Z Mei H Gao S Liang C Whole-genome and whole-exome sequencing of bladder cancer identifies frequent alterations in genes involved in sister chromatid cohesion and segregation Nat Genet 2013 45 1459 1463 10.1038/ng.2798 24121792 PMC7512009

[B33] Balbas-Martinez C Sagrera A Carrillo-de-Santa-Pau E Earl J Marquez M Vazquez M Lapi E Castro-Giner F Beltran S Bayes M Carrato A Cigudosa JC Dominguez O Gut M Herranz J Juanpere N Kogevinas M Langa X Lopez-Knowles E Lorente JA Lloreta J Pisano DG Richart L Rico D Salgado RN Tardon A Chanock S Heath S Valencia A Losada A Recurrent inactivation of STAG2 in bladder cancer is not associated with aneuploidy Nat Genet 2013 45 1464 1469 10.1038/ng.2799 24121791 PMC3840052

[B34] Kagey MH Newman JJ Bilodeau S Zhan Y Orlando DA van Berkum NL Ebmeier CC Goossens J Rahl PB Levine SS Taatjes DJ Dekker J Young RA Mediator and cohesin connect gene expression and chromatin architecture Nature 2010 467 430 435 10.1038/nature09380 20720539 PMC2953795

[B35] Dixon JR Selvaraj S Yue F Kim A Li Y Shen Y Hu M Liu JS Ren B Topological domains in mammalian genomes identified by analysis of chromatin interactions Nature 2012 485 376 380 10.1038/nature11082 22495300 PMC3356448

[B36] Lieberman-Aiden E van Berkum NL Williams L Imakaev M Ragoczy T Telling A Amit I Lajoie BR Sabo PJ Dorschner MO Sandstrom R Bernstein B Bender MA Groudine M Gnirke A Stamatoyannopoulos J Mirny LA Lander ES Dekker J Comprehensive mapping of long-range interactions reveals folding principles of the human genome Science 2009 326 289 293 10.1126/science.1181369 19815776 PMC2858594

[B37] Li Y Huang W Niu L Umbach DM Covo S Li L Characterization of constitutive CTCF/cohesin loci: a possible role in establishing topological domains in mammalian genomes BMC Genomics 2013 14 553 10.1186/1471-2164-14-553 23945083 PMC3765723

[B38] Seitan V Faure A Zhan Y McCord R Lajoie B Ing-Simmons E Lenhard B Giorgetti L Heard E Fisher A Flicek P Dekker J Merkenschlager M Cohesin-based chromatin interactions enable regulated gene expression within pre-existing architectural compartments Genome Res 2013 23 2066 2077 10.1101/gr.161620.113 24002784 PMC3847776

[B39] Sofueva S Yaffe E Chan W-C Georgopoulou D Vietri Rudan M Mira-Bontenbal H Pollard SM Schroth GP Tanay A Hadjur S Cohesin-mediated interactions organize chromosomal domain architecture EMBO J 2013 32 3119 3129 10.1038/emboj.2013.237 24185899 PMC4489921

[B40] Zuin J Dixon JR van der Reijden MI Ye Z Kolovos P Brouwer RW van de Corput MP van de Werken HJ Knoch TA van Ijcken WF Grosveld FG Ren B Wendt KS Cohesin and CTCF differentially affect chromatin architecture and gene expression in human cells Proc Natl Acad Sci U S A 2014 111 996 1001 10.1073/pnas.1317788111 24335803 PMC3903193

[B41] Stedman W Kang H Lin S Kissil JL Bartolomei MS Lieberman PM Cohesins localize with CTCF at the KSHV latency control region and at cellular c-myc and H19/Igf2 insulators EMBO J 2008 27 654 666 10.1038/emboj.2008.1 18219272 PMC2262040

[B42] Wendt KS Yoshida K Itoh T Bando M Koch B Schirghuber E Tsutsumi S Nagae G Ishihara K Mishiro T Yahata K Imamoto F Aburatani H Nakao M Imamoto N Maeshima K Shirahige K Peters JM Cohesin mediates transcriptional insulation by CCCTC-binding factor Nature 2008 451 796 801 10.1038/nature06634 18235444

[B43] Parelho V Hadjur S Spivakov M Leleu M Sauer S Gregson HC Jarmuz A Canzonetta C Webster Z Nesterova T Cobb BS Yokomori K Dillon N Aragon L Fisher AG Merkenschlager M Cohesins Functionally Associate with CTCF on Mammalian Chromosome Arms Cell 2008 132 422 433 10.1016/j.cell.2008.01.011 18237772

[B44] Faure AJ Schmidt D Watt S Schwalie PC Wilson MD Xu H Ramsay RG Odom DT Flicek P Cohesin regulates tissue-specific expression by stabilizing highly occupied cis-regulatory modules Genome Res 2012 22 2163 2175 10.1101/gr.136507.111 22780989 PMC3483546

[B45] Schmidt D Schwalie P Ross-Innes CS Hurtado A Brown G Carroll J Flicek P Odom D A CTCF-independent role for cohesin in tissue-specific transcription Genome Res 2010 20 578 588 10.1101/gr.100479.109 20219941 PMC2860160

[B46] Dorsett D Merkenschlager M Cohesin at active genes: a unifying theme for cohesin and gene expression from model organisms to humans Curr Opin Cell Biol 2013 25 3 327 333 10.1016/j.ceb.2013.02.003 23465542 PMC3691354

[B47] Kawauchi S Calof AL Santos R Lopez-Burks ME Young CM Hoang MP Chua A Lao T Lechner MS Daniel JA Nussenzweig A Kitzes L Yokomori K Hallgrimsson B Lander AD Multiple organ system defects and transcriptional dysregulation in the Nipbl(+/-) mouse, a model of Cornelia de Lange Syndrome PLoS Genet 2009 5 e1000650 10.1371/journal.pgen.1000650 19763162 PMC2730539

[B48] Liu J Feldman R Zhang Z Deardorff MA Haverfield EV Kaur M Li JR Clark D Kline AD Waggoner DJ Das S Jackson LG Krantz ID SMC1A expression and mechanism of pathogenicity in probands with X-Linked Cornelia de Lange syndrome Hum Mutat 2009 30 1535 1542 10.1002/humu.21095 19701948 PMC2783874

[B49] Dorsett D Eissenberg JC Misulovin Z Martens A Redding B McKim K Effects of sister chromatid cohesion proteins on cut gene expression during wing development in Drosophila Development 2005 132 4743 4753 10.1242/dev.02064 16207752 PMC1635493

[B50] Schaaf CA Misulovin Z Sahota G Siddiqui AM Schwartz YB Kahn TG Pirrotta V Gause M Dorsett D Regulation of the Drosophila Enhancer of split and invected-engrailed gene complexes by sister chromatid cohesion proteins PLoS ONE 2009 4 e6202 10.1371/journal.pone.0006202 19587787 PMC2703808

[B51] Zhou Y Kurukuti S Saffrey P Vukovic M Michie AM Strogantsev R West AG Vetrie D Chromatin looping defines expression of TAL1, its flanking genes and regulation in T-ALL Blood 2013 122 4199 4209 10.1182/blood-2013-02-483875 24200685

[B52] Gao X Johnson KD Chang YI Boyer ME Dewey CN Zhang J Bresnick EH Gata2 cis-element is required for hematopoietic stem cell generation in the mammalian embryo J Exp Med 2013 210 2833 2842 10.1084/jem.20130733 24297994 PMC3865483

[B53] Diffner E Beck D Gudgin E Thoms JA Knezevic K Pridans C Foster S Goode D Lim WK Boelen L Metzeler KH Micklem G Bohlander SK Buske C Burnett A Ottersbach K Vassiliou GS Olivier J Wong JW Gottgens B Huntly BJ Pimanda JE Activity of a heptad of transcription factors is associated with stem cell programs and clinical outcome in acute myeloid leukemia Blood 2013 121 2289 2300 10.1182/blood-2012-07-446120 23327922

[B54] Wang Q Stacy T Binder M Marin-Padilla M Sharpe AH Speck NA Disruption of the Cbfa2 gene causes necrosis and hemorrhaging in the central nervous system and blocks definitive hematopoiesis Proc Natl Acad Sci U S A 1996 93 3444 3449 10.1073/pnas.93.8.3444 8622955 PMC39628

[B55] Okuda T van Deursen J Hiebert SW Grosveld G Downing JR AML1, the target of multiple chromosomal translocations in human leukemia, is essential for normal fetal liver hematopoiesis Cell 1996 84 321 330 10.1016/S0092-8674(00)80986-1 8565077

[B56] Schlegelberger B Gohring G Thol F Heuser M Update on cytogenetic and molecular changes in myelodysplastic syndromes Leuk Lymphoma 2012 53 525 536 10.3109/10428194.2011.618235 21877899

[B57] Horsfield JA Anagnostou SH Hu JK Cho KH Geisler R Lieschke G Crosier KE Crosier PS Cohesin-dependent regulation of Runx genes Development 2007 134 2639 2649 10.1242/dev.002485 17567667

[B58] Nottingham WT Jarratt A Burgess M Speck CL Cheng JF Prabhakar S Rubin EM Li PS Sloane-Stanley J Kong ASJ de Bruijn MF Runx1-mediated hematopoietic stem-cell emergence is controlled by a Gata/Ets/SCL-regulated enhancer Blood 2007 110 4188 4197 10.1182/blood-2007-07-100883 17823307 PMC2234795

[B59] Ng CE Yokomizo T Yamashita N Cirovic B Jin H Wen Z Ito Y Osato M A Runx1 intronic enhancer marks hemogenic endothelial cells and hematopoietic stem cells Stem Cells 2010 28 1869 1881 10.1002/stem.507 20799333

[B60] Marsman J O’Neill AC Kao BR Rhodes JM Meier M Antony J Monnich M Horsfield JA Cohesin and CTCF differentially regulate spatiotemporal runx1 expression during zebrafish development Biochim Biophys Acta 1839 2014 50 61 10.1016/j.bbagrm.2013.11.007 24321385

[B61] Li G Ruan X Auerbach RK Sandhu KS Zheng M Wang P Poh HM Goh Y Lim J Zhang J Sim HS Peh SQ Mulawadi FH Ong CT Orlov YL Hong S Zhang Z Landt S Raha D Euskirchen G Wei CL Ge W Wang H Davis C Fisher-Aylor KI Mortazavi A Gerstein M Gingeras T Wold B Sun Y Extensive promoter-centered chromatin interactions provide a topological basis for transcription regulation Cell 2012 148 84 98 10.1016/j.cell.2011.12.014 22265404 PMC3339270

[B62] Panigrahi AK Pati D Higher-order orchestration of hematopoiesis: is cohesin a new player? Exp Hematol 2012 40 967 973 10.1016/j.exphem.2012.09.010 23022223 PMC3595174

[B63] Yan J Enge M Whitington T Dave K Liu J Sur I Schmierer B Jolma A Kivioja T Taipale M Taipale J Transcription factor binding in human cells occurs in dense clusters formed around cohesin anchor sites Cell 2013 154 801 813 10.1016/j.cell.2013.07.034 23953112

