# Supplementary material for: Cohesin mutations in myeloid malignancies: underlying mechanisms
Source: Exp Hematol Oncol. 2014 May 8;3:13. doi: 10.1186/2162-3619-3-13 (PMC4046106; doi:10.1186/2162-3619-3-13)
Supplement: Additional file 1: Table S1 — Comprehensive summary of cohesin mutations in myeloid malignancies. [file 2162-3619-3-13-S1.docx]

**Additional file 1: Table S1: Comprehensive summary of cohesin mutations in myeloid malignancies.**

| **Citation** | **Details of study**  Number of AML samples  (Type of sample)  *Sequencing method* | **Cohesin subunit(s) mutated or deleted in AML**  Occurrence/ total samples (% frequency) | **Karyotype or cytogenetic classification of cohesin-mutated samples*** | **Principle findings** |
| --- | --- | --- | --- | --- |
| Walter MJ, Payton JE, Ries RE, Shannon WD, Deshmukh H, Zhao Y, Baty J, Heath S, Westervelt P, Watson M a, Tomasson MH, Nagarajan R, O’Gara BP, Bloomfield CD, Mrózek K, Selzer RR, Richmond T a, Kitzman J, Geoghegan J, Eis PS, Maupin R, Fulton RS, McLellan M, Wilson RK, Mardis ER, Link DC, Graubert T a, DiPersio JF, Ley TJ: **Acquired copy number alterations in adult acute myeloid leukemia genomes.** *Proc Natl Acad Sci U S A* 2009, **106**:12950–5. | 86 (AML patient-paired bone marrow and skin samples)  *SNP array and comparative genomic hybridisation* | ***STAG2***  1/86 (1.2%) | Cytogenetically normal | Genome-wide copy number analysis was performed to identify alterations in AML genomes not detected by classical cytogenetics.  40% of AML genomes contained copy number alterations (CNAs) not found by classical cytogenetics. Most CNAs detected encompassed known AML-associated genes.  One karyotypically normal patient was found to have a 57-kb deletion on the X chromosome, encompassing the *STAG2* gene. |
| Bullinger L, Krönke J, Schön C, Radtke I, Urlbauer K, Botzenhardt U, Gaidzik V, Carió a, Senger C, Schlenk RF, Downing JR, Holzmann K, Döhner K, Döhner H: **Identification of acquired copy number alterations and uniparental disomies in cytogenetically normal acute myeloid leukemia using high-resolution single-nucleotide polymorphism analysis.** *Leukemia* 2010, **24**:438–49 | 67 (AML patient bone marrow or blood samples)  *Microarray* | ***RAD21***  1/67 (1.5%) | Cytogenetically normal | Genome-wide analysis of single nucleotide polymorphisms (SNPs) was performed in cytogenetically normal AML (CN-AML) samples, to detect microdeletions and acquired uniparental disomy (UPD) not detected by classical cytogenetics.  12% of cases displayed acquired UPDs, and 49% of cases had acquired CNAs.  One case was found to have a deletion of 8q23.3-24.11, containing the *RAD21* gene. |
| Rocquain J, Gelsi-Boyer V, Adélaïde J, Murati A, Carbuccia N, Vey N, Birnbaum D, Mozziconacci M-J, Chaffanet M: **Alteration of cohesin genes in myeloid diseases**. *Am J Hematol* 2010, **85**:717–719 | 167 (AML, myelodysplastic syndrome and chronic myelomonocytic leukemia samples-tissue unspecified)  *Array comparative genomic hybridisation* | ***STAG2***  1/167 (0.6%)  ***RAD21***  1/167 (0.6%)  **Total cohesin**  2/167 (1.2%) | Karyotypically normal | Array-comparative genomic hybridisation (aCGH) was performed on 167 myeloid disorder samples with normal karyotypes, including AML, myelodysplastic syndromes (MDS), and chronic myelomonocytic leukemias (CMML). Two-thirds of samples showed no aCGH copy number alterations.  One AML patient was found to harbor a deletion at Xq25, resulting in a loss of the *STAG2* gene. Deletion of *RAD21* was detected in one CMML patient who subsequently developed M5 FAB AML.  Aneuploidy was not detected in either sample, suggesting that it is cohesin’s non-mitotic roles that are important for leukemogenesis. |
| Ding L, Ley TJ, Larson DE, Miller C a, Koboldt DC, Welch JS, Ritchey JK, Young M a, Lamprecht T, McLellan MD, McMichael JF, Wallis JW, Lu C, Shen D, Harris CC, Dooling DJ, Fulton RS, Fulton LL, Chen K, Schmidt H, Kalicki-Veizer J, Magrini VJ, Cook L, McGrath SD, Vickery TL, Wendl MC, Heath S, Watson M a, Link DC, Tomasson MH, et al.: **Clonal evolution in relapsed acute myeloid leukaemia revealed by whole-genome sequencing.** *Nature* 2012, **481**:506–10 # | 8 (AML patient-paired diagnosis and relapse samples plus matched skin controls)  *Whole-genome sequencing*  200 (AML data from TCGA dataset)  *In silico screening* | ***SMC3***  1/8 (12.5%)  ***SMC3***  6/200 (3%)  **Total cohesin**  7/208 (3.4%) | Karyotypically normal | The genomes of primary and relapse tumors were sequenced to identify the mutational spectrum of primary versus relapse AML. In addition to known AML genes (e.g *FTL3, NPM1*), several novel mutations were identified, including *WAC, DIS3, DDX41,* *DAXX,* and *SMC3.*  To validate that these genes were truly recurrently mutated in AML, an *in silico* screen of TCGA AML data was performed. A further 6 cases of *SMC3* mutation were identified.  Two patterns of clonal evolution leading to relapse were identified; one where the founding clone of the primary tumor gains mutations and evolves into the relapse clone; a second pattern where a subclone of the founder survives therapy, gains mutations, and proliferates at relapse. |
| Walter MJ, Shen, Dong, Ding L, Shao J, Koboldt DC, Chen K, Larson DE, McLellan MD, Dooling D, Abbott R, Fulton R, Magrini V, Schmidt H, Kalicki-Veizer J, Laughlin M, Fan X, Grillot M, Witowski S, Heath S, Frater JL, Eades W, Tomasson M, Westervelt P, DiPersio JF, Link DC, Mardis ER, Ley TJ, Wilson RK, Graubert TA: **Clonal architecture of secondary acute myeloid leukemia**. *New Engl J Med* 2012, **366**:1090–8 | 7 (secondary -AML patient-matched bone marrow samples plus skin controls)  *Whole-genome sequencing*  7 (samples from the preceding MDS stage in the same patients)  *Targeted sequencing of the mutations identified by WGS* | ***STAG2***  1/7 (14.3%)  ***SMC3***  1/7 (14.3%)  **Total cohesin**  2/7 (28.6%)  ***STAG2***  1/7 (14.3%)  ***SMC3***  1/7 (14.3%)  **Total cohesin**  2/7 (28.6%) | Both karyotypically normal  Both karyotypically normal | In order to study the development of secondary-AML from MDS, bone marrow samples and matched skin controls from seven secondary AML patients were submitted to WGS. By comparing the mutations found in each sample to those identified in the TCGA AML project, recurrent mutations were distinguished from background mutations.  A STAG2 mutation was detected in one patient, and an SMC3 mutation was detected in another. Both these mutations were also present in the MDS sample, and the karyotype was normal in both samples at both stages.  The proportion of neoplastic bone marrow was the same in MDS and secondary-AML samples, suggesting that MDS are as clonal as AML. Clonal analysis showed that the founding clone of all seven MDS samples persisted in the secondary-AML samples. However secondary-AML samples were not monoclonal, because of the acquisition of further mutations by diversifying daughter clones. This suggested that progression from MDS to AML could be driven by particular daughter clones, not merely by the recurrent founder mutations. |
| Greif PA, Dufour A, Konstandin NP, Ksienzyk B, Zellmeier E, Tizazu B, Sturm J, Benthaus T, Herold T, Yaghmaie M, Dörge P, Hopfner K-P, Hauser A, Graf A, Krebs S, Blum H, Kakadia PM, Schneider S, Hoster E, Schneider F, Stanulla M, Braess J, Sauerland MC, Berdel WE, Büchner T, Woermann BJ, Hiddemann W, Spiekermann K, Bohlander SK: **GATA2 zinc finger 1 mutations associated with biallelic CEBPA mutations define a unique genetic entity of acute myeloid leukemia.** *Blood* 2012, **120**:395–403 | 5 (AML patient bone marrow or peripheral blood)  *Whole-exome sequencing* | ***STAG2***  1/5 (20%) | Normal karyotype | In order to identify potential cooperator mutations in cytologically normal bi*CEPBA*-mutated AML, Whole-exome sequencing was performed in five bi*CEPBA* AML cases.  A *STAG2* mutation was identified in one patient.  *GATA2* mutations were detected in 2/5 cases; when followed by targeted sequencing of further bi*CEPBA* cases, *GATA2* mutations were detected in 39.4% of cases. No further cohesin mutations were detected. |
| Huh J, Kim H-J, Jung CW, Kim H-J, Kim S-H, Kim Y-K, Kim H-J, Shin MG, Moon JH, Sohn SK, Kim SH, Lee WS, Won JH, Mun YC, Kim H, Park J, Min WS, Kim DHD: **A genome-wide single-nucleotide polymorphism-array can improve the prognostic stratification of the core binding factor acute myeloid leukemia.** *Am J Hematol* 2012, **87**:961–8 | 98 (core binding factor AML (CBF AML) patient bone marrow samples)  *Whole genome SNP analysis* | ***RAD21***  1/98 (1%) | Cytogenetically abnormal. | To understand the prognostic value of SNP analysis in CBF AML, the genomes of 98 CBF AML patients were analyzed using the Affymetrix Human SNP 6.0 array in comparison to metaphase cytogenetics.  One patient was found to harbor an 8q24 deletion encompassing *RAD21*.  Overall, the findings suggested that SNP analysis in combination with metaphase cytogenetics was a useful prognostic tool. |
| Welch JS, Ley TJ, Link DC, Miller C a, Larson DE, Koboldt DC, Wartman LD, Lamprecht TL, Liu F, Xia J, Kandoth C, Fulton RS, McLellan MD, Dooling DJ, Wallis JW, Chen K, Harris CC, Schmidt HK, Kalicki-Veizer JM, Lu C, Zhang Q, Lin L, O’Laughlin MD, McMichael JF, Delehaunty KD, Fulton L a, Magrini VJ, McGrath SD, Demeter RT, Vickery TL, et al.: **The origin and evolution of mutations in acute myeloid leukemia.** *Cell* 2012, **150**:264–78 # | 24 (AML patient-paired bone marrow and skin samples)  *Whole-genome sequencing*  84 (Additional AML patient samples)  *Targeted resequencing of candidate genes identified in the original 24 cancers*  183 (Additional AML samples, TCGA dataset)  *In silico screening of cohesin genes* | ***SMC3***  1/24 (4.2%)  ***SMC1A***  1/24 (4.2%)  ***STAG2***  1/24 (4.2%)  ***SMC3***  1/84 (1.2%)  ***SMC1A***  1/84 (1.2%)  ***STAG2***  2/84 (2.4%)  **Total cohesin**  7/108 (6.5 %)  **Total cohesin**  19/183 (10.4%) | All karyotypically normal | To understand clonal evolution in AML, the genomes of normal karyotype M1-AML samples without a known initiating event and M3-AML samples with a known initiating event (*PML-RARA)* were sequenced, as were the exomes of HSCs from healthy people.  Sequencing the HSCs of healthy people showed that these cells accumulate benign mutations throughout life. In combination with the finding that number of mutations in AML samples correlates with the age of the patient, this suggested that most mutations in AML are passengers.  Sequencing of the M1 and M3 AML genomes revealed genes that are recurrently mutated in AML, and are therefore likely to be causal mutations. Nine genes were recurrently mutated in both M1 and M3 genomes, while 13 were recurrently mutated in only M1 genomes. As *PML-RARA* is the initiating event in M3 AML, the nine recurrent mutations are likely to be co-operative, rather than initiating mutations. In contrast, the 13 genes recurrently mutated in only M1 genomes, which included *SMC1A, SMC3,* and *STAG2*, are more likely to be important to AML initiation.  Cohesin mutation co-occurred with *NPM1, TET2, DNMT3A,* or *RUNX1* mutations in the majority of cases. Mutations in cohesin components were mutually exclusive, suggesting that a mutation in one subunit is sufficient to disrupt the entire complex. Cohesin mutations were only ever found in M1 genomes, and were not associated with chromosomal instability, suggesting that cohesin mutation is an early event in oncogenesis, and that roles other than chromosome cohesion are responsible for cohesin’s involvement in AML. |
| Jan M, Snyder TM, Corces-Zimmerman MR, Vyas P, Weissman IL, Quake SR, Majeti R: **Clonal evolution of preleukemic hematopoietic stem cells precedes human acute myeloid leukemia.** *Sci. Transl. Med.* 2012;4(149):149-118. † | 6 (AML patient blood samples)  *Exome sequencing*  120 (Genomic DNA samples) *Targeted sequencing of SMC1A* | ***SMC1A***  2/6 (33.3%)  ***SMC1A***  1/120 (0.83%)  **Total *SMC1A***  3/126 (2.4%) | Both karyotypically normal  Data unavailable | In order to identify the initiating mutations important to the development of AML, exome sequencing was performed on 6 *de novo* AML samples harboring *FLT*-ITD mutations, and residual hematopoeitic stem cells (HSCs) from the same patients.  57 genes were mutated over the 6 AML exomes, including common AML mutations like *FLT3, IDH1, NPM1* and *TET2.* Two of the six cases harbored *SMC1A* mutations. Targeted sequencing in an additional 120 AML samples identified one further *SMC1A* mutation.  Several of the identified mutations (including *SMC1A*) were also found at varying frequencies in the patients’ residual HSCs, identifying these cells as preleukemic HSCs. Single-cell analysis of preleukemic HSCs revealed the serial acquisition of mutations during clonal evolution.  In the *SMC1A*-mutated cases, *TET2* was the founding mutation, with *SMC1A* and a secondary *TET2* mutation following in the dominant preleukemic clone. Interestingly, in another sample without *SMC1A* mutations, *TET2* founder mutations were followed by *CTCF* mutations.  This suggested a model where serial mutations accumulate in self-renewing HSCs, allowing progression to AML. Residual HSCs harboring preleukemic mutations may continue to accumulate mutations post-treatment, resulting in relapse. |
| Dolnik A, Engelmann JC, Scharfenberger-Schmeer M, Mauch J, Kelkenberg-Schade S, Haldemann B, Fries T, Krönke J, Kühn MWM, Paschka P, Kayser S, Wolf S, Gaidzik VI, Schlenk RF, Rücker FG, Döhner H, Lottaz C, Döhner K, Bullinger L: **Commonly altered genomic regions in acute myeloid leukemia are enriched for somatic mutations involved in chromatin remodeling and splicing.** *Blood* 2012, **120**:e83–92 | 50 (AML patient-paired diagnosis and remission blood and/or bone marrow samples)  *Targeted re-sequencing of candidate loci identified in* [1–3]  120 (AML patient blood and/or bone marrow samples)  *Targeted sequencing of RAD21* | ***RAD21***  3/50 (6%)  ***RAD21***  4/120 (3.33%)  **Total *RAD21***  7/170 (4.1%) | 4 karyotypically abnormal  3 karyotypically normal | To identify new mutations in AML, 1000 genes located in regions recurrently altered in AML were sequenced in a representative sample cohort (n=50) representing cytologically normal AML, complex karyotype AML and core-binding factor AML.  60 non-silent single nucleotide variants (SNVs), and 125 indels were identified over the 50 cases. Geneset analysis revealed an enrichment of epigenetic regulators among the mutated genes, including chromatin modification, chromosome organization, and histone modification.  Three out of fifty cases harbored a *RAD21* mutation. To explore the incidence of mutations in *RAD21* in AML, *RAD21* coding exons 2-14 were screened in an additional 120 AML patients. This identified 4 further cases with *RAD21* mutations.  *RAD21* mutations were not restricted to any particular subgroup of AML, but were significantly more frequent in patients with *RAS* mutations. |
| Walter MJ, Shen D, Shao J, Ding L, White BS, Kandoth C, Miller C a, Niu B, McLellan MD, Dees ND, Fulton R, Elliot K, Heath S, Grillot M, Westervelt P, Link DC, DiPersio JF, Mardis E, Ley TJ, Wilson RK, Graubert T a: **Clonal diversity of recurrently mutated genes in myelodysplastic syndromes.** *Leukemia* 2013, **27**:1275–82 | 8 (*de novo* MDS and paired secondary-AML patient samples)  *Whole-genome sequencing*  150 (*de novo* MDS patients samples)  *Targeted sequencing of 94 MDS candidate genes* | ***STAG2***  1/8 (12.5%)  ***STAG2***  9/150 (6%)  ***SMC3***  2/150 (1.3%)  **Total cohesin**  12/158 (7.6%) | Normal karyotype  6 normal karyotype  5 abnormal karyotype | Following the 2012 study by the same authors, the mutational status of additional MDS samples was interrogated by sequencing, to further define the clonal architecture and founding mutations of MDS.  Samples from eight *de novo* MDS patients who subsequently developed secondary-AML were submitted to whole-genome sequencing.  A further 150 *de novo* MDS cases were sequenced for a panel of 94 MDS candidate genes. Mutations identified in the eight-sample WGS were included in the panel, as were various MDS-associated genes from the literature, and AML-associated genes from the TCGA AML study [4].  Mutations in cohesin subunits *STAG2* and *SMC3* were identified. The mutational status of the other cohesin subunits remains unknown in this cohort, as these genes were not included in the 94-gene panel.  The eight secondary-AML genomes were found to be oligoclonal: made up of one founding clone and several daughter clones. Each clone contained at least one mutation that is recurrent in MDS/AML.  Mutations in *RUNX1* and *STAG2, BCOR* and *U2AF1* co-occurred more frequently than predicted by chance. |
| The Cancer Genome Atlas Research Group: **Genomic and Epigenomic Landscapes of Adult De Novo Acute Myeloid Leukemia**. *N Engl J Med* 2013, **368**:2059–2074 † # | 200 (AML patient-paired tumour and skin samples; TCGA dataset)  *Whole-genome sequencing (50 samples), exome sequencing (150 samples)* | ***SMC3***  7/200 (3.5%)  ***SMC1A***  7/200 (3.5%)  ***SMC5***  1/200 (0.5%)  ***STAG2***  6/200 (3%)  ***RAD21***  5/200 (2.5%)  **Total cohesin**  26/200 (13%) | 17 karyotypically normal  9 abnormal karyotypes  1 data unavailable | 200 samples from adult *de novo* AML patients representing the major cytogenetic subtypes of AML were submitted to genome/exome sequencing, with the goal of identifying novel genomic variants of importance to the development of AML.  The mutations identified were grouped into functional categories with a putative link to AML pathogenesis: transcription factor fusions, *NPM1* mutations, tumor-suppressors, DNA-methylation and chromatin modification genes, myeloid transcription factors, spliceosome complex genes, and the genes of the cohesin complex.  Cohesin-complex mutations were generally mutually exclusive. Interestingly, mutations in myeloid transcription factors, *ASLX1* mutations, and mutations of epigenetic modifiers were also generally mutually exclusive with cohesin mutations.  Cohesin mutations significantly co-occurred with *NPM1, DNMT3A, FLT3,* and *PTP5.* |
| Kon A, Shih L-Y, Minamino M, Sanada M, Shiraishi Y, Nagata Y, Yoshida K, Okuno Y, Bando M, Nakato R, Ishikawa S, Sato-Otsubo A, Nagae G, Nishimoto A, Haferlach C, Nowak D, Sato Y, Alpermann T, Nagasaki M, Shimamura T, Tanaka H, Chiba K, Yamamoto R, Yamaguchi T, Otsu M, Obara N, Sakata-Yanagimoto M, Nakamaki T, Ishiyama K, Nolte F, et al.: **Recurrent mutations in multiple components of the cohesin complex in myeloid neoplasms.** *Nat Genet* 2013, **45**:1232–7 † | 610 (various myeloid neoplasms)  *Targeted sequencing of cohesin complex genes. SNP arrays for copy number alterations at cohesin loci.*  157 (acute myeloid leukemia)  224 (Myelodysplastic syndromes)  88 (chronic myelomonocytic leukemia)  64 (chronic myelogenous leukemia)  77 (classical myeloproliferative neoplasms) | **Total cohesin**  65/610 (10.7%)  ***SMC3***  1/157 (0.64%)  ***SMC1A***  2/157 (1.3%)  ***STAG2***  11/157 (7%)  ***RAD21***  7/157 (4.5%)  ***NIPBL***  1/157 (0.63%)  ***PDS5***  1/157 (0.63%)  **Total cohesin**  23/157 (14.6%)  ***SMC3***  3/224 (1.3%)  ***STAG2***  14/224 (6.25%)  ***RAD21***  2/224 (0.9%)  ***NIPBL***  1/224 (0.45%)  ***STAG1***  1/224 (0.45%)  ***ESCO2***  1/224 (0.45%)  **Total cohesin**  22/224 (9.8%)  ***STAG2***  9/88 (10.2%)  ***PDS5***  1/88 (1.1%)  ***STAG1***  1/88 (1.1%)  ***ESCO2***  1/88 (1.1%)  **Total cohesin**  12/88 (13.6%)  ***SMC1A***  2/64 (3.1%)  ***STAG2***  2/64 (3.1%)  ***RAD21***  1/64 (1.6%)  ***NIPBL***  1/64 (1.6%)  **Total cohesin**  5/64 (7.8%) ^§^  ***STAG2***  1/77 (1.3%)  ***PDS5***  1/77 (1.3%)  **Total cohesin**  2/77 (2.6%) | 27 karyotypically normal  28 karyotypically abnormal  10 data unavailable | In order to investigate a role for cohesin in myeloid leukemogenesis, the cohesin genes were sequenced in 610 primary specimens of various myeloid neoplasms.  Four cohesin genes were found to be significantly mutated in myeloid neoplasms: *SMC3, SMC1A, STAG2,* and *RAD21.*  In addition, four of 34 myeloid leukemia cell lines surveyed harbored cohesin mutations, and forced expression of wild-type cohesin subunits produced growth suppression in two of these cohesin-depleted lines (Kasumi-1 and MOLM-13).  In the various cohesin-depleted cell lines, expression of cohesin subunits in chromatin-bound fractions was reduced in comparison to whole-cell extract.  Cohesin mutations were mostly mutually exclusive, and showed significant association with mutations in *TET2, ASXl1,* and *EZH2.*  Deep sequencing of mutant alleles in 20 available samples revealed the allele frequency of these mutations in the tumor population. In 15/20 cases, the cohesin mutation was present in the major tumour population, indicating that cohesin mutations often occur as early events in leukemogenesis.  There was no significant difference in chromosome abnormalities between cohesin-mutant and cohesin-wild-type samples, with all cases showing diploid or near-diploid karyotypes. |
| Yoshida K, Toki T, Okuno Y, Kanezaki R, Shiraishi Y, Sato-Otsubo A, Sanada M, Park M, Terui K, Suzuki H, Kon A, Nagata Y, Sato Y, Wang R, Shiba N, Chiba K, Tanaka H, Hama A, Muramatsu H, Hasegawa D, Nakamura K, Kanegane H, Tsukamoto K, Adachi S, Kawakami K, Kato K, Nishimura R, Izraeli S, Hayashi Y, Miyano S, et al.: **The landscape of somatic mutations in Down syndrome-related myeloid disorders.** *Nat Genet* 2013, **45**:1293–9 | 4 (Patient-matched transient abnormal myelopoeisis (TAM), acute megakaryoblastic leukemia (AMKL) and remission samples)  *Whole-genome sequencing*  14 (Down Syndrome AMKL samples)  *Exome sequencing*  49 (DS-AMKL)  19 (non-DS-AMKL)  *Targeted deep sequencing of genes identified in whole genome/exome sequenced samples, as well as other genes frequently mutated in AMKL.* | ***STAG2***  ¼ (25%)  ***RAD21***  2/4 (50%)  **Total cohesin**  3/4 (75%)  ***STAG2***  3/14 (21.4%)  ***RAD21***  4/14 (28.6%)  ***NIPBL***  1/14 (7.1%)  **Total cohesin**  8/14 (57.1%)  **Total cohesin mutated/deleted in DS-AMKL**  26/49 (53%)  **Total cohesin mutated in non-DS-AMKL**  2/19 (11%) | 12 karyotypically abnormal with trisomy 21  5 otherwise normal with trisomy 21  9 data unavailable  2 karyotypically abnormal with trisomy 21 | To investigate the pathogenesis of Down syndrome (DS) related myeloid disorders, the genomes of transient abnormal myelopoiesis (TAM), DS-associated acute megakaryoblastic leukemia (DS-AMKL), and non-DS-AMKL samples were sequenced.  TAM development seems to result from *GATA1* mutation plus constitutive trisomy 21. Subsequently, progression to DS-AMKL seems to require the acquisition of further mutations.  Mutational targets in the progression to DS-AMKL were identified as recurrently mutated genes in DS-AMKL: *RAD21,* *STAG2*, *NRAS, CTCF, DCAF7, EZH2, KANSL1,* and *TP53.*  Cohesin mutations were mutually exclusive, and more frequent in DS-AMKL than non-DS-AMKL.  Cohesin mutations were not associated with a significant difference in aneuploidy.  Mutations in cohesin had comparable allelic burden to *GATA1* mutations in this cohort, suggesting their role is also in early stages of DS-AMKL development. |
| Kandoth C, McLellan MD, Vandin F, Ye K, Niu B, Lu C, Xie M, Zhang Q, McMichael JF, Wyczalkowski M a, Leiserson MDM, Miller C a, Welch JS, Walter MJ, Wendl MC, Ley TJ, Wilson RK, Raphael BJ, Ding L: **Mutational landscape and significance across 12 major cancer types.** *Nature* 2013, **502**:333–9 # | 200 (AML data from TCGA dataset)  *Whole genome/exome sequencing* | ***SMC3***  7/200 (3.5%)  ***SMC1A***  7/200 (3.5%)  ***STAG2***  6/200 (3%)  ***RAD21***  5/200 (2.5%)  **Total cohesin**  26/200 (13%) | TCGA dataset, therefore as in [4] | The mutational landscape of 12 cancer types was assessed using genome/exome sequence generated as part of the TCGA dataset.  Cohesin mutations were prevalent in AML (13%), as well as in bladder urothelial carcinoma, uterine corpus endometrial carcinoma, and lung adenocarcinoma. |
| Thol F, Bollin R, Gehlhaar M, Walter C, Dugas M, Suchanek KJ, Kirchner A, Huang L, Chaturvedi A, Wichmann M, Wiehlmann L, Shahswar R, Damm F, Göhring G, Schlegelberger B, Schlenk R, Döhner K, Döhner H, Krauter J, Ganser A, Heuser M: **Mutations in the cohesin complex in acute myeloid leukemia: clinical and prognostic implications.** *Blood* 2013 † | 389 (AML patient bone marrow or peripheral blood)  *Targeted sequencing of cohesin complex genes* | ***SMC3***  5/389 (1.3%)  ***SMC1A***  2/389 (0.5%)  ***STAG1***  7/389 (1.8%)  ***STAG2***  5/389 (1.3%)  ***RAD21***  4/389 (1%)  **Total cohesin**  23/389 (5.9%) | 15 karyotypically normal  7 karyotypically abnormal  1 data unavailable | Following previous reports of cohesin mutations in AML, targeted sequencing of cohesin complex genes was performed in 389 AML samples, to examine the frequency of cohesin as a mutational target in AML. Correlation to clinical data was included to assess the clinical and prognostic implications of cohesin mutations.  A total of 23 mutations were found in the cohesin complex (5.9%). Mutations in cohesin subunits were mutually exclusive, and most mutations were found in karyotypically normal samples.  A strong correlation was observed between cohesin and *NPM1* mutations, with *NPM1*-mutated patients twice as likely to also harbor a cohesin mutation compared to *NPM1*-wildtype patients.  Cohesin mutation status was not found to be prognostically informative, nor was it correlated with any differences in clinical features.  Allelic burden analysis suggested that cohesin mutations occurred as early event during leukemogenesis. |
| Lawrence MS, Stojanov P, Mermel CH, Robinson JT, Garraway L a., Golub TR, Meyerson M, Gabriel SB, Lander ES, Getz G: **Discovery and saturation analysis of cancer genes across 21 tumour types**. *Nature* 2014 # | 196 (AML data from TCGA dataset)  *Whole genome/exome sequencing* | ***SMC3***  7/196 (3.6%)  ***SMC1A***  7/196 (3.6%)  ***STAG2***  6/196 (3.1%)  ***RAD21***  6/196 (3.1%)  **Total cohesin**  26/196 (13.3%) | TCGA dataset, therefore as in [4] | Exome sequencing was carried out in 4742 human cancer samples, across 21 cancer types, with the goal of finding mutations common to many cancers, and creating a comprehensive catalogue of cancer genes.  The experimental approach was able to identify nearly all known cancer genes, and in addition, 33 novel genes were identified as significantly mutated in cancer.  Rad21 was identified as one of the set of ‘novel’ cancer genes in the all-tumours cohort.  In the cohort of 196 AML samples, *SMC3*, *STAG2*, and *RAD21* were each mutated approximately 3%. In total there were 13.3% cohesin complex mutations in this AML cohort. |
| Pellagatti A, Fernandez-Mercado M, Di Genua C, Larrayoz MJ, Killick S, Dolatshad H, Burns A, Calasanz MJ, Schuh A, Boultwood J: **Whole-exome sequencing in del(5q) myelodysplastic syndromes in transformation to acute myeloid leukemia.** *Leukemia* 2013:1–4. | 3 (MDS patients before and after progression to AML)  *Whole-exome sequencing* | ***SMC1B***  1/3 (33.3%) | Karyotypically abnormal | In order to better understand the molecular mechanisms of MDS progression to AML, whole-exome sequencing was undertaken in 3 MDS patients before and after progression to AML.  A mutation in meiotic cohesin component *SMC1B* was found in one patient. The mutation was present in both pre-AML and AML samples.  Recurrent mutations were noted in *TP53* and *RYR1.* |
| Haferlach T, Nagata Y, Grossmann V, Okuno Y, Bacher U, Nagae G, Schnittger S, Sanada M, Kon a, Alpermann T, Yoshida K, Roller a, Nadarajah N, Shiraishi Y, Shiozawa Y, Chiba K, Tanaka H, Koeffler HP, Klein H-U, Dugas M, Aburatani H, Kohlmann a, Miyano S, Haferlach C, Kern W, Ogawa S: **Landscape of genetic lesions in 944 patients with myelodysplastic syndromes.** *Leukemia* 2013, **28**:241–247 | 944 (myelodyplastic syndrome (MDS) bone marrow/peripheral blood samples)  *Targeted sequencing of 104 known/putative gene targets. Copy-number analysis by aCGH.* | **Total cohesin**  Data unavailable (Approximately 15%) | Data unavailable | Sequencing of 104 known/putative mutations was carried out in a cohort of 944 MDS patients, and survival was analyzed in 875 patients, to develop a molecular profiling system for classification and prognostics in MDS.  47 genes were significantly mutated in MDS, 25 of which were significantly associated with survival outcome.  A combination of 14 of these genes with traditional classification systems produced a prognostic model with four risk-groups (low, intermediate, high and very high risk) predicting different survival outcomes. A second prognostic model utilizing the 14 genes alone recapitulated the four risk-groups, indicating that profiling of genetic targets is useful for classification and prognosis of MDS.  Sequencing results revealed that approximately 15% of MDS harbor cohesin complex mutations. *STAG2* and *SMC1A* were both significantly associated with worse survival outcome. *STAG2* was included in the gene-only prognostic model. |
| Kihara R, Nagata Y, Kiyoi H, Kato T, Yamamoto E, Suzuki K, Chen F, Asou N, Ohtake S, Miyawaki S, Miyazaki Y, Sakura T, Ozawa Y, Usui N, Kanamori H, Kiguchi T, Imai K, Uike N, Kimura F, Kitamura K, Nakaseko C, Onizuka M, Takeshita a, Ishida F, Suzushima H, Kato Y, Miwa H, Shiraishi Y, Chiba K, Tanaka H, et al.: **Comprehensive analysis of genetic alterations and their prognostic impacts in adult acute myeloid leukemia patients**. *Leukemia* 2014(February):1–44. | 197 (AML patient bone marrow samples)  *Targeted sequencing of the exons of 51 genes recurrently mutated in myeloid neoplasms.* | ***SMC3***  5/197 (2.5%)  ***SMC1A***  4/197 (2%)  ***STAG2***  10/197 (5.1%)  ***RAD21***  4/197 (2%)  **Total cohesin**  23/197 (11.7%) | 8 karyotypically normal  14 karyotypically abnormal  1 data unavailable | An analysis of the association between mutation status and clinical/prognostic features was undertaken in 197 cases of AML, aiming to refine the risk classification system for AML by adding mutation status to traditional cytogenetics.  The analysis demonstrated that AML patients could be stratified into five groups for overall survival if mutation status for *DNMT3A, MLL-*PTD, and *TP53* were combined with the current European LeukemiaNet system.  11.7% of this cohort possessed cohesin complex mutations. Although cohesin mutations were not associated with rate of complete remission or of disease-free survival, patients with cohesin mutations displayed improved overall survival compared to patients without cohesin mutations. |

* Where specified by original study.

† Data used to produce Figure 1.

# Study used TCGA data.

§ One CML case harboring both *STAG2* and *SMC1A* mutations was counted as a single case.

**Supplementary References**

1. Bullinger L, Krönke J, Schön C, Radtke I, Urlbauer K, Botzenhardt U, Gaidzik V, Carió a, Senger C, Schlenk RF, Downing JR, Holzmann K, Döhner K, Döhner H: **Identification of acquired copy number alterations and uniparental disomies in cytogenetically normal acute myeloid leukemia using high-resolution single-nucleotide polymorphism analysis.** *Leukemia* 2010, **24**:438–49.

2. Rücker FG, Schlenk RF, Bullinger L, Kayser S, Teleanu V, Kett H, Habdank M, Kugler C-M, Holzmann K, Gaidzik VI, Paschka P, Held G, von Lilienfeld-Toal M, Lübbert M, Fröhling S, Zenz T, Krauter J, Schlegelberger B, Ganser A, Lichter P, Döhner K, Döhner H: **TP53 alterations in acute myeloid leukemia with complex karyotype correlate with specific copy number alterations, monosomal karyotype, and dismal outcome.** *Blood* 2012, **119**:2114–21.

3. Rücker FG, Bullinger L, Schwaenen C, Lipka DB, Wessendorf S, Fröhling S, Bentz M, Miller S, Scholl C, Schlenk RF, Radlwimmer B, Kestler H a, Pollack JR, Lichter P, Döhner K, Döhner H: **Disclosure of candidate genes in acute myeloid leukemia with complex karyotypes using microarray-based molecular characterization.** *J Clin Oncol* 2006, **24**:3887–94.
